# 90552 Evidence synthesis with reconstructed survival data

**DOI:** 10.1017/cts.2021.519

**Published:** 2021-03-30

**Authors:** Chenqi Fu, Shouhao Zhou, Nicholas Short, Xuelin Huang, Donald Berry, Farhad Ravandi-Kashani

**Affiliations:** 1 Pennsylvania State University; 2 MD Anderson Cancer Center

## Abstract

ABSTRACT IMPACT: A one-stage Bayesian multilevel model for meta-analysis integrating different survival data is introduced to complete the information synthesis without assuming proportional hazard. OBJECTIVES/GOALS: To develop a general modeling approach to perform efficient and robust meta-analyses using aggregated data (AD) for survival type endpoint and apply to a meta-analysis to examine the association between measurable residual disease (MRD) and disease-free survival (DFS) and overall survival (OS) in patients with acute myeloid leukemia (AML). METHODS/STUDY POPULATION: A Bayesian semi-parametric hierarchical model with a time-varying HR effect was presented. Three common types of survival information, including reconstructed survival data, the hazard ratio (HR) estimates with corresponding CIs and survival rates at specific time points, are synthesized such that all literature from the systematic review can contribute properly to the estimation and uncertainty quantification of the model parameters. The time-varying effects was modeled by assuming piecewise hazard risk and piecewise constant hazard ratio. The heterogeneity across studies was expressed by an additive random study effect and a random treatment-by-study interaction. The method was applied to a systematic review of 81 publications reporting on 11,151 AML patients. RESULTS/ANTICIPATED RESULTS: In simulation studies that the proportional hazard assumption is either valid or violated, the proposed method was efficient to achieve comparable performance to IPD meta-analysis, a gold standard approach, in estimating the survival rates, the restricted mean survival time at specific time points and median survival time with the point estimates close to the true values. When HR is not proportional over time, the proposed method was robust in estimating HR and significantly outperformed the classical random-effects meta-analysis. In the application to AML study, the average HR for achieving MRD negativity was 0.36 (95% CrI, 0.33-0.39) for OS and 0.37 (95% CrI, 0.34-0.40) for DFS. The association of MRD negativity with OS and DFS was significant and consistent across all subgroups. DISCUSSION/SIGNIFICANCE OF FINDINGS: The proposed novel method provided a flexible framework for meta-analysis of survival data, to accommodate various types of survival data in one model without assuming proportional hazards assumption. The findings of AML meta-analysis suggest that achievement of MRD negativity is associated with superior DFS and OS in patients with AML.

